# Long term metabolic and renal outcomes of kidney donors compared to controls with excellent kidney function

**DOI:** 10.1186/s12882-019-1214-4

**Published:** 2019-01-31

**Authors:** Ayelet Grupper, Yoel Angel, Aharon Baruch, Idit F. Schwartz, Doron Schwartz, Richard Nakache, Yaacov Goykhman, Paulina Katz, Ido Nachmany, Nir Lubezky, Talia Weinstein, Moshe Shashar, Orit Kliuk Ben-Bassat, Shlomo Berliner, Ori Rogowski, David Zeltser, Itzhak Shapira, Shani Shenhar-Tsarfaty

**Affiliations:** 1 Organ Transplantation Unit, Tel Aviv Sourasky Medical Center and Sackler Faculty of Medicine, Tel Aviv University, 6th Weizman St, 6423906 Tel Aviv, Israel; 2 Nephrology Department, Tel-Aviv Sourasky Medical Center and Sackler Faculty of Medicine, Tel-Aviv University, 6th Weizman St, 6423906 Tel Aviv, Israel; 3 Department of Internal Medicine “C”, “D” and “E”, Tel Aviv Sourasky Medical Center and Sackler Faculty of Medicine, Tel Aviv University, Tel Aviv, Israel; 4 Renal Section, Sanz Medical Center, Laniado Hospital, Netanya, Israel

**Keywords:** Living kidney donor, Hypertension, Albuminuria, eGFR, Metabolic syndrome

## Abstract

**Background:**

Only few studies of living kidney donors have included controls that were similarly healthy, including excellent kidney function.

**Methods:**

In this study, we aimed to estimate long term metabolic and renal outcome in a cohort of 211 living donors compared to two control groups: paired-matched controls, and another control group of 2534 healthy individuals with excellent kidney function.

**Results:**

Donors presented with higher estimated Glomerular Filtration Rate (eGFR): (97.6 ± 15.2 vs 96.1 ± 12.2 vs 94.5 ± 12.4 ml/min/1.73m^2^) and lower urine albumin to creatinine ratio (UACR) (4.3 ± 5.9 vs 5.9 ± 6.1 vs 6.1 ± 6.9 mg/g) for donors, matched controls and healthy controls, respectively (*p* <  0.001). In a mean follow up period of 5.5 for donors, donors presented with positive eGFR slopes during the first 3 years post donation, followed by negative slopes, compared to constantly negative slopes presented in the control group (*p* <  0.05). The variables related to the slope were being a donor, baseline eGFR, Body Mass Index (BMI) and age but not eGFR on the last day of follow-up or increased delta UACR. There was a significant increase in UACR in donors, as well as a higher rate of albuminuria, associated with a longer time since donation, higher pre-donation UACR and higher pre-donation BMI. Healthy controls had a lower BMI at baseline and gained less weight during the follow up period. Donors and controls had similar incidence of new onset diabetes mellitus and hypertension, as well as similar delta systolic and diastolic blood pressure. Donors were more likely to develop new onset metabolic syndrome, even after adjustment for age, gender and BMI. The higher incidence of metabolic syndrome resulted mainly from increased triglycerides and impaired fasting glucose criteria. However, prevalence of major cardiovascular events was not higher in this group.

**Conclusions:**

Donors are at increased risk to develop features of the metabolic syndrome in addition to the expected mild reduction of GFR and increased urine albumin excretion. Future studies are needed to explore whether addressing those issues will impact post donation morbidity and mortality.

## Background

Reduced kidney function is associated with increased risk of mortality and cardiovascular morbidity in the general population [1, 2]. Nephrectomy, for the purpose of kidney donation, inevitably leads to reduced renal mass and function [3] and is associated with an increased proteinuria, as well as a rise in blood pressure (BP), greater than that attributable to normal aging [4, 5]. Consequently, there is a concern that reduced Glomerular Filtration Rate (GFR) following nephrectomy will have a direct impact on donors’ subsequent health, in addition to the potential indirect effects of a reduced GFR that could accelerate other post-donation events associated with aging, such as type 2 diabetes mellitus (DM) or hypertension (HTN).

Available data on long term risks following kidney donation vary depending on the selected control group, with few studies showing that live donors have a better outcome than their population counterparts, while other studies presenting increased risks such as End-Stage Renal Disease (ESRD), HTN and cardiovascular mortality [6–10].

In these studies, risk assessment was done either by comparison of kidney donors to the general population or to matched controls, which were selected from national or local registries or from medical claims data. Each of these approaches has its limitations [11, 12] but conclusions have thus far been limited by lack of appropriate comparison groups, reliance on insurance claims, or ascertainment bias. An appropriate control group should include healthy individuals selected in a manner comparable with the screening process for kidney donors, including specifically superb kidney function.

A better knowledge of major metabolic and renal outcomes in living kidney donors is essential for the choice of potential donors, and in order to guide long term follow-up care to maintain good health.

In this study, we aimed to estimate long term metabolic consequences and renal outcome in a stratum of living donors and two control groups: healthy individuals with excellent kidney function composed the first, and the second group was comprised of matched controls by demographic, medical and kidney function parameters.

## Methods

### Study populations

Living donors: included in the study are 215 consecutive live kidney donors who donated a kidney between January 2000 and January 2016 in Tel Aviv Sourasky Medical Center, with at least 1 year of follow up post donation. All donors underwent a comprehensive evaluation before donation, according to the local protocols, and routine follow up post donation at the kidney transplantation unit. Excluded from the study are four donors who were lost to follow up.

**Fig. 1:**
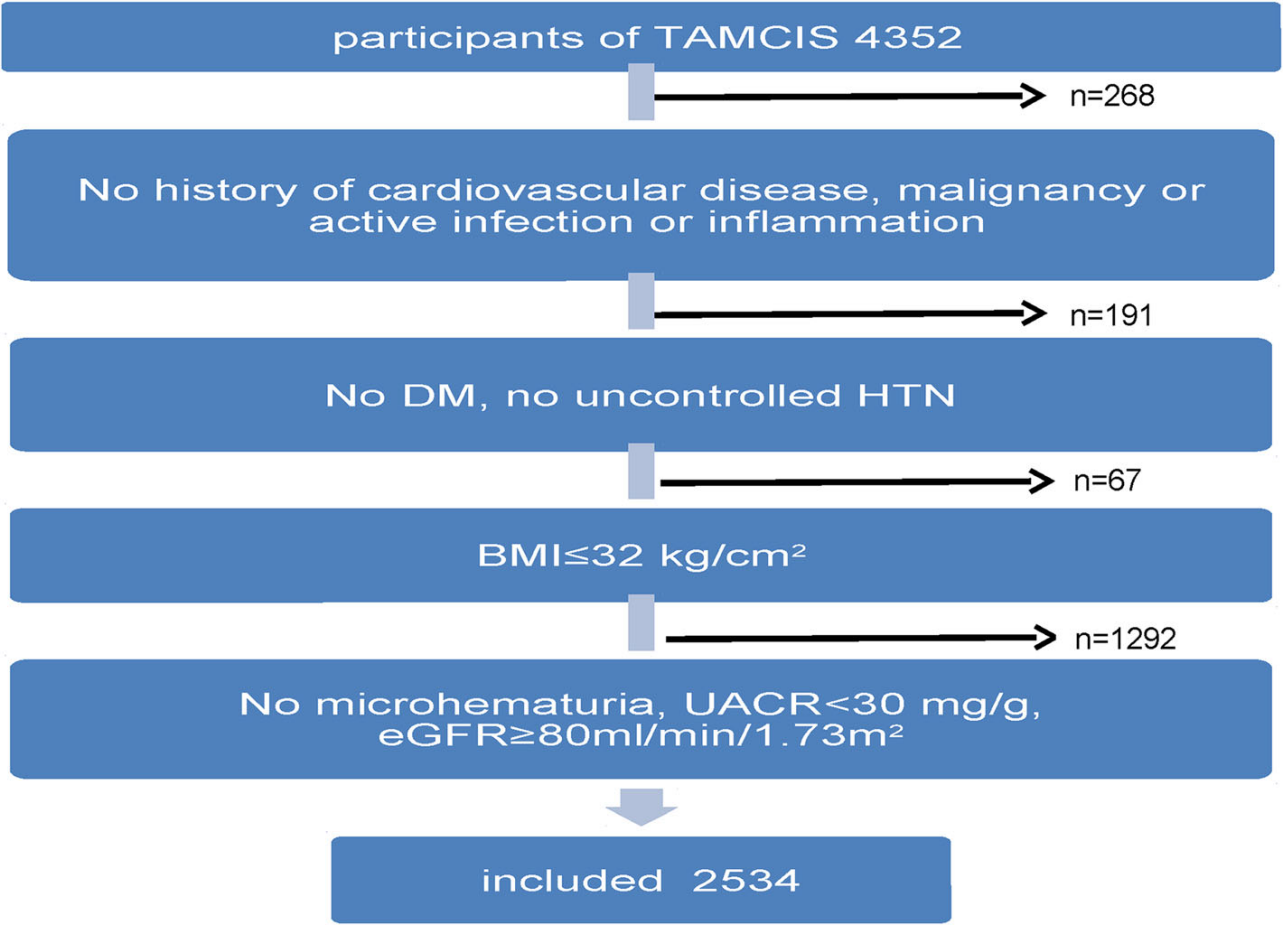
Algorithm for participants exclusion from the TAMCIS (Tel Aviv Medical Center Inflammatory Survey) to cohort of healthy controls. DM, diabetes mellitus; HTN, hypertension; UACR, Urine Albumin to Creatinine Ratio

Cohort of healthy controls: The control cohort was analyzed using the database of the Tel Aviv Medical Center Inflammation Survey (TAMCIS), a registered databank which encompass a large cohort of subjects who attended our medical center for routine annual checkup examination between 2002 and 2016 (including a physician’s interview and examination, blood and urine tests). For the purpose of our study we identified participants younger than 70 years old, with at least 4 consecutive visits. We excluded participants with any of the following conditions:

Active cancer, infection or inflammation; cardiovascular disease; DM; uncontrolled HTN (BP > 140/90 mmHg on two or more antihypertensive medications, or use of more than two antihypertensive medications); Body Mass Index (BMI) ≥ 32 kg/m^2^; evidence of kidney disease: microhematuria (more than 3 RBC/hpf), estimated GFR (eGFR) < 80 ml/min/1.73m^2^, urine Albumin to Creatinine Ratio (UACR) > 30 mg/g (Fig. 1). At the end of the exclusion process, 2534 individuals were included in the healthy control group.

The matched controls: from the aforementioned control group, we used Semi-Automatic Matching Platform (SAMPL), a computerized tool developed at our institution, for selection of paired matched controls. The matching was on a 1:1 ratio, and based on a set of the following criteria:

Length of Follow up (± 6 months), eGFR (±10 ml/min/1.73m^2^), age (± 2 years), gender (same), BMI (± 1 kg/m^2^) and current smoking status (same).

For each pair of donor and possible control, the SAMPL tool calculated a “dissimilarity score” based on the differences in values for the above criteria. For example, if the donor and control had exactly the same age, BMI, eGFR. etc. then the dissimilarity score would be “0”; as differences accumulated the dissimilarity score increased. For every donor, the control with the lowest dissimilarity score (i.e., minimum differences in matching criteria) was chosen to be the matched – control.

A written informed consent was obtained from the control participants and the two studies were approved by the local ethics committee (number 0738–16 and 02–049).

### Laboratory and clinical parameters

Blood samples were drawn after a 12-h overnight fast. The ratio of glycated hemoglobin (A1C) and total hemoglobin were measured and reported as % A1C. Hemoglobin A1C (HbA1C) levels were categorized into three: healthy: < 5.7%; pre-diabetic: 5.7–6.4% and diabetic: > 6.5% according to the American Diabetes Association (ADA) guidelines [13].

Metabolic syndrome was defined according to NCEP ATP III, requires the presence of any three of the following five traits: BP > 130/85 mmHg (or drug treatment for HTN); serum triglycerides (TG) ≥150 (or drug treatment for hypertriglyceridemia); Serum High Density Lipoprotein (HDL) cholesterol < 40 mg/dL in men and < 50 mg/dL in women or drug treatment for low HDL cholesterol; Abdominal obesity (a waist circumference in men ≥102 cm and in women ≥88 cm); Fasting plasma glucose≥100 mg/dL or drug treatment for elevated blood glucose [14, 15].

Those criteria were changed according to our available data: HbA1C > 5.7% or treatment with hypoglycemic medications was used to define pre-diabetes [16]; BMI > 30 kg/m^2^ was used to define obesity [17].

GFR was estimated (eGFR) using the Chronic Kidney Disease Epidemiology Collaboration (CKD-EPI) creatinine equation, a 4-variable formula [18] adjusted for Body Surface Area (Mosteller calculation).

Urine albumin was measured as urine albumin to creatinine ratio (UACR) determined from a spot urine sample. Moderately increased albuminuria (category A2, formerly microalbuminuria) was defined as UACR between 30 to 300 mg/g [19].

Resting BP was measured in triplicates in the nondominant arm using a validated oscillometric sphygmomanometer after 15 min of rest. The mean of the last two measurements was used for analysis. HTN was defined as BP > 140/90 mmHg or the use of antihypertensive medications.

### Statistical analysis

All data was summarized and displayed as mean ± Standard Deviation (SD) for the continuous variables and as number of patients and the percentage in each group for categorical variables. For all categorical variables, the Chi-Square statistic was used to assess the statistical significance between groups.

Continuous variables were first tested for normal distribution by using a Kolmogorov-Smirnov test and Q-Q plots, then parameters were compared by using a t test or by Kruskal Wallis/Mann-Whitney test.

We fitted binary logistic regression models for a eGFR< 60 ml/min/1.73m^2^, moderately increased albuminuria and HTN, adjusted for age at donation, gender, time since donation, BMI, smoking status, and baseline (pre-donation for donors, first visit for controls) eGFR.

For the matched control group, paired analysis was performed for comparison of eGFR, systolic and diastolic BP, and UACR differences.

The evaluation of new diagnosed DM and metabolic syndrome, and adjusting for potential confounders, was performed with cox regression analysis, Adjusted for gender, age, family history of DM and BMI.

The date of visit at which the end-points were diagnosed was considered as the time-point for the regression analysis.

Delta of a specific parameter defined as the value measured on Last Day of Follow Up (LDFU) minus the baseline (pre-donation for donors, first visit for controls) value.

eGFR slope was calculated as delta eGFR divided by the time interval between the visits and represented as ml/min/1.73m^2^/year.

*P* < 0.05 was considered statistically significant for all analyses.

IBM SPSS Statistics for Windows, version 22 (IBM Corp., Armonk, N.Y., USA) was used for all statistical analysis.

## Results

Participants’ demographic, clinical characteristics and laboratory data of the study groups are summarized in Table 1. All participants were Caucasians. About half of the donors were living related (121/211, 57%). Compared to healthy controls, the donors group was characterized by lower percentage of males and higher BMI. The matched-control group was superior to the “healthy controls” group in that it showed excellent similarity in all reported baseline characteristics.

**Table 1:** Baseline population characteristics of kidney donors, matched controls, and healthy control group

Parameter, mean (SD)	Donors	Matched controls	*p* value	Healthy controls	*P* value
n	211	211		2534	
Age, years	42.3 (12.0)	42.3 (11.6)	0.125	43.6 (8.9)	0.153
Male gender, %	55.9	55.9	1	66.8	< 0.001
BMI, kg/m^2^	25.6 (3.4)	25.3 (3.3)	0.995	25 (2.7)	0.006
Current smokers, %	26.9	24.1	0.417	20.8	0.059
eGFR ml/min/1.73m^2^	97.6 (15.2)	96.1 (12.2)	< 0.001	94.5 (12.4)	< 0.001
UACR, mg/g	4.3 (5.9)	5.9 (6.1)	0.008	6.1 (6.9)	< 0.001
Systolic BP, mmHg	121.6 (11.5)	117.5 (13.3)	0.06	119.6 (13.7)	0.128
Diastolic BP, mmHg	74.2 (8.0)	73.9 (7.1)	0.88	75.5 (7.8)	0.029
HTN, %	10.9	10.7	0.841	12.8	0.449
Pre Diabetes, %	10	12.8	0.194	9.1	0.246
Metabolic Syndrome, %	5.2	3.8	0.639	3.3	0.162
DM family history, %	28.4	28.2	0.97	35.3	0.19
Follow Up Period, y	5.5 (3.7)	5.4 (3.0)	0.39	5.3 (1.1)	0.366

*BMI* body mass index, *BP* blood pressure, *DM* diabetes mellitus, *eGFR* estimated GFR, *HTN* hypertension, *SD* standard deviation, *UACR* urine albumin to creatinine ratio

Donors presented with higher eGFR and lower UACR compared to both control groups.

Age range pre-donation (first visit) was 19.9–69.1, 20.1–68.9, and 21–70.1 years for donors, matched controls and healthy controls, respectively, with a similar mean. The mean age on LDFU was 48.56 ± 9.1, 48.91 ± 10.7, and 47.1 ± 12.8 years for donors, matched controls and healthy controls, respectively (*p* = 0.17). The mean follow-up period was similar with a range of 1–16 years in all study groups.

Three donors died during the follow up period, all from cancer (squamous cell carcinoma, leukemia, and thyroid carcinoma). None of the healthy and matched-control groups died during the study period.

### Kidney function: During the first 3 years post donation positive eGFR slope was observed in donors, compared to negative slope thereafter and in controls

**Fig. 2:**
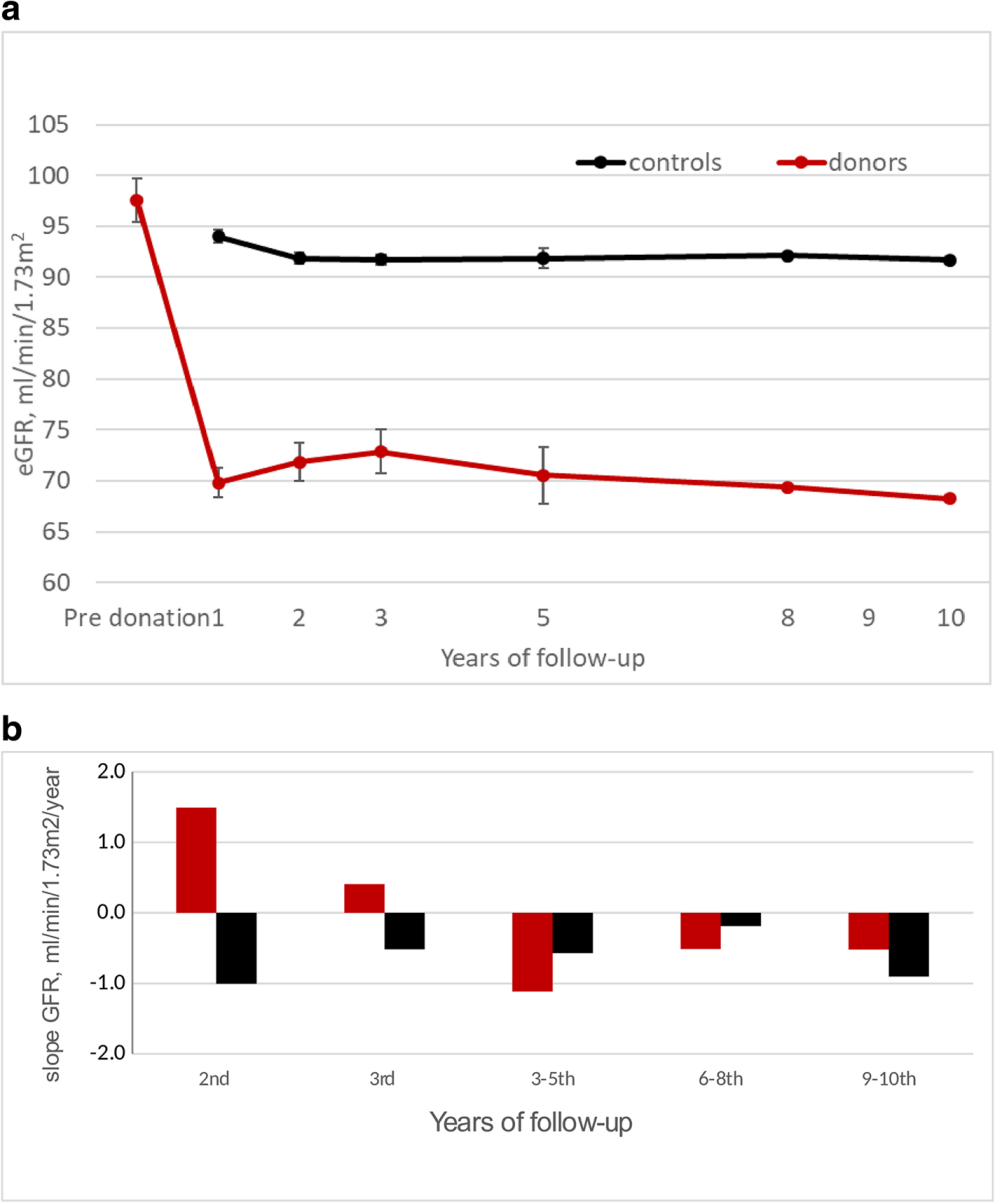
Change of estimated Glomerular Filtration Rate (eGFR) with time. **a** Mean eGFR in the donors’ and control groups. **b** Annual eGFR slope of donors’ and control groups. In the donors group, eGFR slopes during the second and third year post donation were positive (1.49 ± 0.79, 0.46 ± 0.48 ml/min/1.73m^2^), and significantly different from the slope in the following years post donation and from controls’ slope, which were negative and constant (*p* < 0.05)

Neither one of the donors nor the control group individuals developed ESRD during the follow up period. The mean eGFR over time for donors and controls is shown in Fig. 2a. In the donors group, eGFR slopes during the second and third year post donation were similar (1.49 ± 0.79, 0.46 ± 0.48 ml/min/1.73m^2^/year in the second and third year, respectively, *p* > 0.05) and significantly different from 3 to 10 years post donation in this group. The controls’ slope was constant through-out the follow up period and similar to the slope 3–10 years post donation in the donors group and significantly different from the first 3 years’ slope of the donors group (*p* < 0.05) Overall *p* value for all groups is 0.007.

The variables related to the slope in the second year were baseline eGFR (*p* < 0.001), BMI (*p* = 0.033), and younger age (*p* = 0.048) in addition to being a donor (*p* < 0.001).

Variables related to the slope in the third to fifth year were baseline eGFR and the slope in the second year (*p* < 0.001 for both).

eGFR on the last day of follow up was related to baseline eGFR, younger age and being a donor (*p* < 0.001 for all) but was not related to slopes of eGFR.

The majority of donors (68.2%) had an eGFR greater than 60 ml/min/1.73m^2^ on LDFU, compared to 90.7% in the healthy controls and 95.7% in the matched control cohort (*p* < 0.001).

None had eGFR lower than 30 ml/min/1.73m^2^. The risk of having an eGFR lower than 60 ml/min/1.73m^2^ was associated with being a donor (Odds Ratio (OR) 12.1 [95% Confidence Interval (CI) 5.1–28.7], *p* < 0.001); male gender (OR 1.541 [95% CI 1.063–2.233], *p* = 0.022), baseline eGFR (OR 0.838 [95% CI 0.818–0.859], *p* < 0.001), age (OR 1.027 [95% CI 1.009–1.045], *p* = 0.003), baseline BMI (OR 1.15 [CI 1.1–1.205], *p* < 0.001).

Type of donor (related vs. unrelated), HTN, UACR (pre-donation or on LDFU), time since donation and smoking status were not significantly associated with this risk.

### Increased risk of albuminuria and increased delta ACR in donors compare to controls

Urine albumin excretion is presented in Table 2. The number of donors with albuminuria at the end of follow-up was higher compared to healthy controls. In addition, a significant increase in UACR was demonstrated in the donors’ group post donation and was not observed in the control group. None of the donors or matched-controls had albuminuria greater than 300 mg/g, while 2 (of 2534) of healthy controls had reached that level.

**Table 2:** Urine albumin excretion in the study cohorts

Parameter, mean (SD)	Donors	Matched-controls	Healthy controls	*P* value
baseline UACR, mg/gr	4.3 (5.9) ^a^	5.9 (6.1) ^b^	6.1 (6.9) ^c^	< 0.001
LDFU UACR, mg/gr	13.5 (26.7) ^a^	7.4 (11.1) ^b^	8.7 (21.9) ^b^	< 0.001
Delta UACR	9.2 (24.6) ^a^	1.62 (13.6) ^b^	3.1 (24.2) ^b^	0.002
Delta UACR> 8 mg/g	28%	14.4%	14.9%	< 0.001
% albuminuria	12.3%	0%	5%	< 0.001

*LDFU* last day of follow up, *SD* standard deviation, *UACR* urine albumin to creatinine ratio

letters annotation mark significant difference between groups in post hoc analysis

The appearance of albuminuria in donors was associated with a longer time since donation (OR 1.39 [95% CI 1.1–1.6], *p* < 0.001), higher pre-donation UACR (OR 1.16 [95% CI 1.08–1.25], *p* < 0.001) and higher pre-donation BMI (OR 1.2 [95% CI 1.07–1.39], *p* = 0.02). The model explained more than 25% of the variance, while eGFR pre or post donation, age, donor status (related), gender, smoking, delta BMI and HbA1C were not significant parameters.

Delta UACR of more than 8 mg/g was present in 28% of the donors compared to only 14% in controls. The risk of worsening UACR by more than 8 mg/g was found in donors (OR 1.523 [95% CI 1.136–1.754], *p* = 0.001); higher delta BMI (OR 1.129 [95% CI 1.036–1.231], *p* = 0.006); and low eGFR on LDFU (OR 0.987 [95% CI 0.975–0.991], *p* = 0.031); neither delta UACR nor albuminuria were related to slopes of eGFR.

### Metabolic outcomes: Being a donor neither increase the risk for HTN, nor to diabtets or metabolic syndrome

Metabolic outcomes of donors and control groups are shown in Table 3.

**Table 3:** Comparison of metabolic parameters at the beginning and at the end of follow up for kidney donors, matched-controls and healthy control group

Variable	Time	Donors	Matched-controls	Healthy controls	*P* value
		211	211	2534	
BMI, kg/m^2^	Baseline	25.64 ± 3.4^a^	25.3 ± 3.3^a^	25 ± 2.7^b^	0.003
	LDFU	26.3 ± 4.19^a^	25.8 ± 3.8^a^	24.6 ± 3.7^b^	< 0.001
	Delta BMI	+ 0.65 ± 1.4^a^	+ 0.41 ± 1.9^a^	−0.3 ± 2.7^b^	< 0.001
HDL, mg/dl	Baseline	51.3 ± 10.2^b^	58.1 ± 14^a^	55.8 ± 14^a^	< 0.001
	LDFU	54.4 ± 13.1^b^	57.3 ± 14.2^a^	55 ± 14.5^a^	< 0.001
	Delta HDL	+ 3.1 ± 13.8^b^	− 0.7 ± 9.3^a^	− 0.6 ± 9.3^a^	< 0.001
LDL, mg/dl	Baseline	109.8 ± 23.2^b^	117.3 ± 32.9^a^	121.6 ± 30^a^	< 0.001
	LDFU	110.5 ± 28.4	111.1 ± 29.6	112.9 ± 27.4	0.340
	Delta LDL	+ 0.73 ± 22.2	− 5.9 ± 26.9^a^	−7.9 ± 28.6^a^	< 0.001
TG, mg/dl	Baseline	111.1 ± 42.4^a^	99.3 ± 57^b^	108.9 ± 62.4^a^	0.025
	LDFU	117.1 ± 66.7^b^	102.2 ± 62.4^a^	106.9 ± 60.3^a^	0.011
	Delta TG	15.9 ± 51.5^b^	5.1 ± 59.5^a^	3.96 ± 63.4^a^	0.019
SBP, mmHg	Baseline	121.6 ± 11.5^a^	117.4 ± 13.3^b^	119.6 ± 13.7^a^	0.008
	LDFU	126.6 ± 12.6^b^	122.2 ± 14.6^a^	123.1 ± 14.8^a^	0.002
	Delta SBP	5.1 ± 12	4.8 ± 11.7	3.7 ± 13.9	0.214
DBP, mmHg	Baseline	74.2 ± 8^a^	73.9 ± 7.1^a^	75.5 ± 7.8^b^	0.001
	LDFU	76.6 ± 7.9	75.4 ± 8.6	76.9 ± 9.3	0.082
	Delta DBP	2.4 ± 8.5	1.55 ± 8.1	1.29 ± 9.5	0.255
HbA1C, %	Baseline	5.3 ± 0.43^b^	5.26 ± 0.4^a^	5.23 ± 0.4^b^	0.002
	LDFU	5.52 ± 0.5	5.5 ± 0.9	5.45 ± 0.46^a^	0.006
	Delta A1C	0.16 ± 0.5	0.19 ± 0.34	0.18 ± 0.26	0.760
Hypertension, %	Baseline	10.9	10.7	12.8	0.491
	New onset	11.8	7.9	10.6	0.311
Metabolic syndrome, %	Baseline	5.2	3.8	3.3	0.216
	New onset	21.9	13.1	15.1	0.032
Diabetes mellitus, %	Baseline	0	0	0	1
	New onset	5.8	3.3	2.5	0.019
MACE, %	LDFU	1.9	0.5	4.9	< 0.001

*BMI* body mass index, *DBP* diastolic blood pressure, *HDL* high density, lipoprotein, *LDFU* last day of follow up, *LDL* low density lipoprotein, *MACE* major adverse cardiovascular events, *SBP* systolic blood pressure, *TG* triglycerides, *UACR* urine albumin to creatinine ratio

letters annotation mark significant difference between groups in post hoc analysis

Healthy controls had a lower BMI at baseline, and gained less weight during the follow up period compared with donors and matched-controls.

Donors had higher TG levels at baseline, and a higher delta TG than both control groups. HDL was lower in donors at baseline on LDFU with a significantly higher delta HDL; LDL was lower in donors at baseline but similar on LDFU in all study groups. HbA1C was statistically higher on baseline and LDFU in donors, but delta HbA1C was similar.

There were similar frequencies of HTN at baseline and new incidence of HTN in donors and controls, before and following adjustment for age, gender and BMI, as well as similar delta systolic and diastolic BP.

**Fig. 3:**
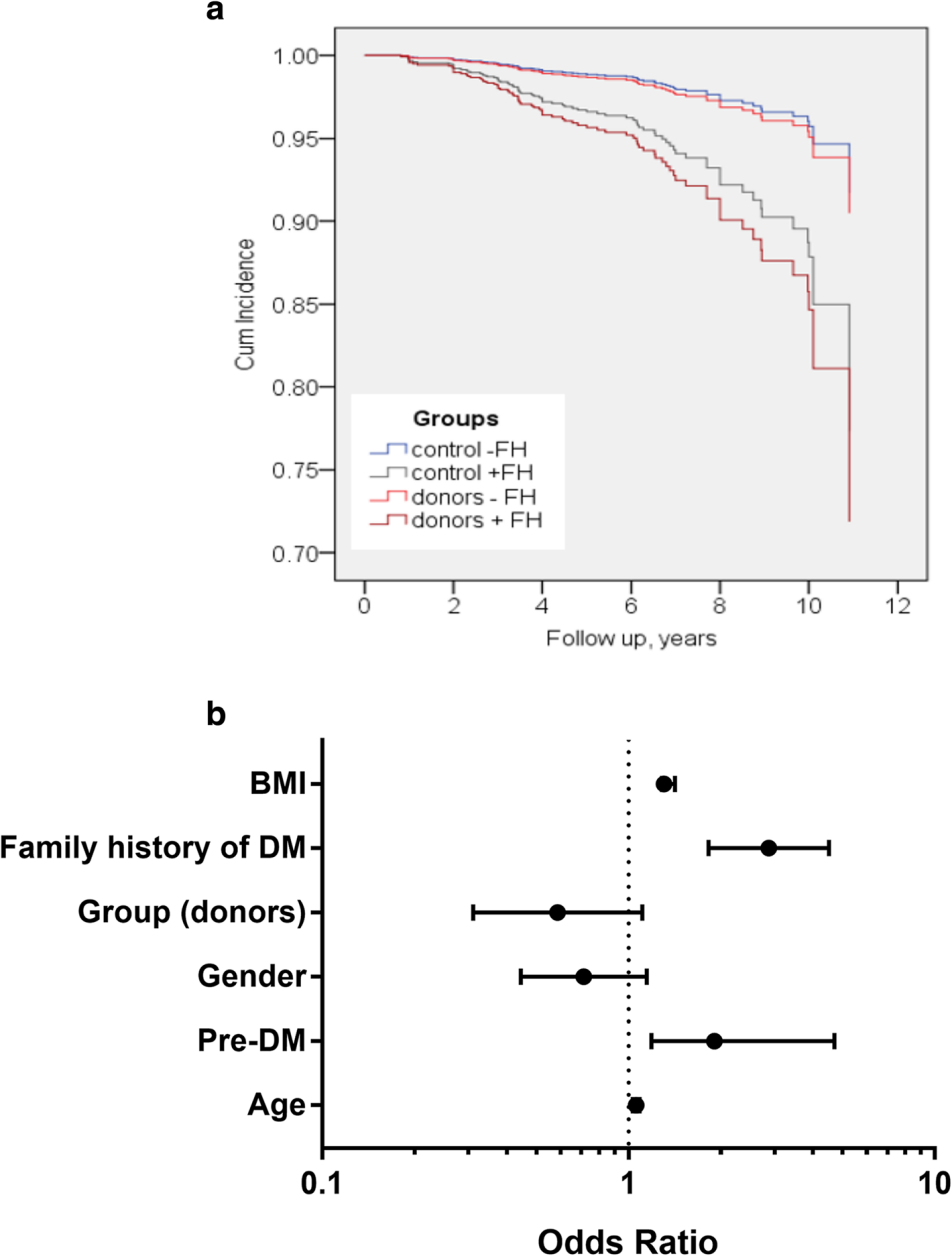
Risk for new onset Diabetes Mellitus (DM). **a** New onset DM according to family history (FH) of diabetes in control/donor groups. Family history of DM significantly increased the risk for new onset DM both in donors and controls (*p* = 0.013) and is more important in determining the risk of new onset DM than being a donor. **b** Forest plot of the risk to develop new onset DM Logistic regression results show that family history, pre-DM age and BMI at baseline are significant predictors for new onset DM (*p* = 0.003 and 0.022, 0.001 and *p* < 0.001 respectively), Note x = 1 (dash line) represent similar risk for new onset DM (odds ratio)

Twelve donors (5.8%) had a new diagnosis of DM compared to 3.3 and 2.5% in the matched and healthy control groups respectively (*p* = 0.019, Table 3). Following adjustment for other confounders (age, gender, BMI, family history and pre-DM), being a donor did not increase the risk for diabetes (*p* = 0.541, Fig. 3a). Baseline Pre-DM and family history of diabetes significantly increased the risk for new onset DM both in donors and controls, (Fig. 3b).

**Fig. 4:**
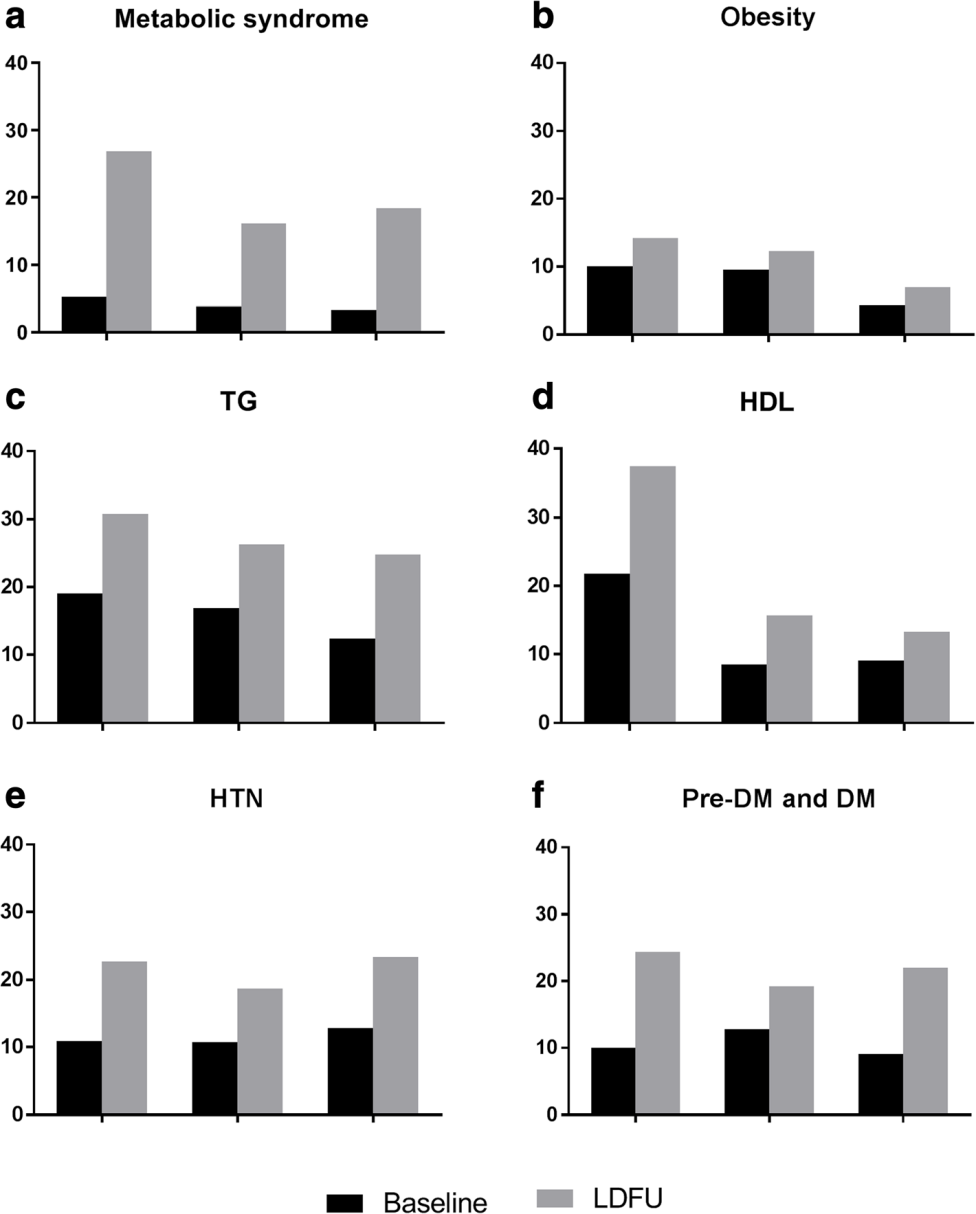
Frequency of metabolic syndrome **a** and each of its criteria **b**-**f** at the start and last day of follow-up (LDFU) of donors and controls. Donors had a higher rate of metabolic syndrome than controls, mainly resulted from the criteria of TG and DM / pre-DM

Donors had a higher rate of new onset metabolic syndrome, after adjustment for age, gender and BMI; The increase of metabolic syndrome in the donors group mainly resulted from the increase of donors fulfilling the TG and impaired fasting glucose criteria. (Fig. 4). However, prevalence of Major Adverse Cardiovascular Events (MACE) was not higher in this group.

## Discussion

Many healthy adults are willing to accept the risks of donor nephrectomy, but the responsibility to quantify those risks lies with the medical community, and efforts should be done to make this information available.

The current study was conducted in order to obtain new tools to better define who are prone to deteriorate following donation and to encourage future studies aimed to prevent the aforementioned process.

Only few studies of living kidney donors have included controls that were equally healthy as donors, including excellent kidney function. In the current study, two groups of controls were utilized: a healthy control group which was composed of healthy individuals with eGFR> 80 ml/min/1.73m^2^ and UACR< 30 mg/g, and matched-controls who had similar demographic and clinical parameters, making them medically comparable to each donor.

A major observation in the current study is the different eGFR pattern over time (slope) post donation, when compared to controls: Donors had a positive slope during the first 3 years post donation, after which it became negative, while the controls’ slope was negative throughout the whole study period, as expected in the general population [20]. The positive slope in our cohort was in correlation to age and BMI, and could to be attributed to compensatory hyperfiltration, as well as to baseline (pre-donation) eGFR. In fact, the increase of eGFR following donation is composed of two phases: An initial rapid increase in GFR which was overlooked in our study, and a second, gradual phase which lasted up to the third year. This finding is with agreement with a previous publication by Kasiske [9]. Much the same, eGFR on last day of follow up was also not related to eGFR changes (slopes) but to older age and baseline eGFR.

The hyperfiltration which was demonstrated 3 years post donation, could reflect a late effect of the nephrectomy, or secondary to the increase of BMI, which were found in the donors group. This hyperfiltration did not correlate with kidney function on last day of follow up, nor to albuminuria or increased urine albumin excretion. Respectively, albuminuria, the hallmark of hyperfiltration damage, did not correlate with eGFR post donation or a change in eGFR.

Our finding about increased risk for moderately increased albuminuria and increased urine albumin excretion are in concordance with previous studies [5].

Moody et al. [8] found that donors had a significant increase in left ventricular mass and mass:volume ratio compared with controls, independently associated with GFR. In addition, donors had global circumferential strain and were more likely to develop highly sensitive troponin T levels and microalbuminuria. Taken together, one can speculate that rather than reflecting glomerular damage, changes in UACR occur due to endothelial damage as part of the metabolic syndrome characteristic of the post donation period.

eGFR less than 60 mL/min/1.73 m^2^ is commonly seen post donation. These values are associated with subsequent morbidity [1]. However, it is uncertain whether these risks apply to donation since in the general population, a low eGFR is a result of kidney or systemic disease, whereas in donors, it is a result of nephrectomy.

Though we utilized GFR estimation by creatinine-based equation, with its limitation [18], in a study of measured GFR by iohexol clearance and estimated GFR in donors and matched control [9], the trend was similar in both and similar to ours during the first 3 years.

Of notice is lack of differences in BP values and in the incidence of HTN between donors and both control groups. Previous studies produced conflicting results [3, 4, 9, 21]. However, only few included well-matched controls. In one of those which did, after 10 years of follow up systolic and diastolic BPs were 6 and 4 mmHg higher respectively in donors than in controls [4]. Garg et al. [21], using claims data, reported that the incidence of HTN was significantly higher in donors than in controls 6.2 years after donation. In contrast, Kasiske et al. did not find any difference between donors and controls during the first 3 years after donation, using ambulatory blood pressure monitoring [9].

Cross-sectional studies suggest that CKD is associated with abnormalities in glucose homeostasis and insulin resistance [22]. Nevertheless, previous studies have not demonstrated any linkage between donation to fasting glucose, hemoglobin A1C, insulin concentrations, or the calculated Homeostasis Model Assessment – Insulin Resistance -HOMA-IR [9].

In the current study, we did not observe a higher incidence of diabetes mellitus or pre-diabetes;

However, we have found a higher incidence of metabolic syndrome in donors compared to controls. One can argue that those differences are related to increased weight gain observed in donors. On the other hand, it may be that donors are encouraged to lose weight before donation, so weight gain post donation should be taken with caution. However, the increase in BMI was similar in matched and donors, therefore we believe that other factors may contribute to our observation. In spite of the differences in rate of new cases of metabolic syndrome, Major Adverse Cardiovascular Events (MACE) rate was not higher in donors.

## Conclusion

Our study has some limitations; first, a major problem in assessing post living kidney donation risk is to find an appropriate controls group, with healthy participants with good kidney function, as the donors are pre-donation. We acknowledge that there will always be limitations in seeking perfect- matched donors. Many potential living kidney donors are screened out after early investigators based upon advanced tests which our matched controls do not have. In order to reduce this bias, we used two control groups: one of 2500 healthy participants with no evidence of kidney disease, and the second consisted with matched controls based on baseline characteristics using the SAMPL methodology.

A second limitation is that the mean follow up period was 6 years, a relatively short period for metabolic outcomes, and prolonged time since donation may unmask differences in additional outcomes parameters related to donation.

Other limitations of our study are a relatively low rate of living donor kidney transplantations and the lack of waist circumference data in order to define the metabolic syndrome criteria.

It would be ideal to be able to determine before donation, at the time of donor evaluation, whether a prospective donor has an increased risk of developing a reduced GFR, HTN, metabolic syndrome or diabetes. Such information may help select donors or counsel prospective donors about their risks and future health care needs.

In summary, we have found that donors are at risk to develop features of the metabolic syndrome and increased urine albumin excretion, in addition to the expected mild reduction of GFR. Future studies are needed to explore whether addressing those issues will impact post donation morbidity and mortality.

## Abbreviations

BMI: Body mass index
BP: Blood pressure
CI: Confidence interval
DM: Diabetes mellitus
eGFR: Estimated Glomerular Filtration Rate
ESRD: End stage renal disease
GFR: Glomerular Filtration Rate
HbA1C: Hemoglobin A1C
HDL: High density lipoprotein
HTN: Hypertension
LDFU: Last Day of Follow Up
LDL: Low density lipoprotein
MACE: Major adverse cardiovascular events
OR: Odds ratio
SD: Standard deviation
TG: Triglycerides
UACR: Urinary albumin to creatinine ratio
